# Identification of HSP90B1 in pan-cancer hallmarks to aid development of a potential therapeutic target

**DOI:** 10.1186/s12943-023-01920-w

**Published:** 2024-01-20

**Authors:** Xiaoliang Huang, Weiming Zhang, Na Yang, Yujie Zhang, Tianyu Qin, Hanyi Ruan, Yan Zhang, Chao Tian, Xianwei Mo, Weizhong Tang, Jungang Liu, Beibei Zhang

**Affiliations:** 1 Division of Colorectal & Anal Surgery, Department of Gastrointestinal Surgery, Guangxi Key Laboratory of Basic and Translational Research for Colorectal Cancer, Guangxi Medical University Cancer Hospital, Nanning, The People’s Republic of China; 2 Department of Clinical Oncology, Wuming Hospital of Guangxi Medical University, Nanning, The People’s Republic of China; 3 Department of Ultrasound, The Second Affiliated Hospital of Kunming Medical University, Kunming, Yunnan The People’s Republic of China; 4 Institute of Biomedical Research, Yunnan University, Kunming, Yunnan The People’s Republic of China

**Keywords:** Pan-cancer, HSP90B1, Immune checkpoint, Immune infiltration, Prognosis

## Abstract

Heat shock proteins play crucial roles in various biochemical processes, encompassing protein folding and translocation. HSP90B1, a conserved member of the heat shock protein family, growing evidences have demonstrated that it might be closely associated with cancer development. In the present study, we employed multi-omics analyses and cohort validations to explore the dynamic expression of HSP90B1 in pan-cancer and comprehensively evaluate HSP90B1 as a novel biomarker that hold promise for precision cancer diagnostics and therapeutics. The results suggest HSP90B1 was highly expressed in various kinds of tumors, often correlating with a poor prognosis. Notably, methylation of HSP90B1 emerged as a protective factor in several cancer types. In immune infiltration analysis, the expression of HSP90B1 in most tumors showed a negative association with CD8 + T cells. HSP90B1 expression was positively correlated with microsatellite instability and tumor mutational burden. HSP90B1 expression was also discovered to be positively correlated with tumor metabolism, cell cycle-related pathways and the expression of immune checkpoint genes. The expression of HSP90B1 was mainly negatively correlated with immunostimulatory genes and positively correlated with immunosuppressive genes, as well as strongly correlated with chemokines and their receptor genes. In addition, the HSP90B1 inhibitor PU-WS13 demonstrated significant efficacy in suppressing cancer cell proliferation in both leukemic and solid tumor cells, and remarkably reduced the expression of the cancer cell surface immune checkpoint PD-L1. The single-cell RNA sequencing analysis further highlighted that HSP90B1 was significantly higher in tumor cells compared to surrounding cells, revealing a potential target therapeutic window. Taken together, HSP90B1 emerges as a promising avenue for breakthroughs in cancer diagnosis, prognosis and therapy. This study provides a rationale for HSP90B1 targeted cancer diagnosis and therapy in future.

**Supplementary Information:**

The online version contains supplementary material available at 10.1186/s12943-023-01920-w.

## Introduction

Cancer stands as a major global public health concern worldwide. Categorized as stress-responsive proteins, heat shock proteins (HSPs) are a group of proteins generated under high temperature induced environment [1]. Facilitated by HSP90, newly formed proteins or proteins under stable stress are folded [2]. HSP90B1, an integral component within the HSP90 family, serves as a chaperone protein localized in the endoplasmic reticulum (ER), which advances the survival of cells in microenvironment by stabilizing and re-folding denatured proteins after pressure-induced challenges [3]. HSP90B1 protein is engaged in impeding both apoptosis and autophagy [4]. Numerous studies have underscored HSP90B1 as a potent molecular adjuvant, functioning as a carrier for tumor antigenic peptides and playing a crucial role in tumor antigen presentation and activation of CD8 + T lymphocytes [5, 6].

HSP90B1 is abundantly deposited in cancer cells and can be bound to tumors [7]. Patients with high expression of HSP90B1 in cancer cells are more susceptible to worse prognosis in comparison to those with low level of expression [8, 9]. Furthermore, HSP90B1 serves as a fundamental immune modulator that influences adaptability and innate immunity. By suppressing inflammatory signaling pathways across various diseases [10], it can enable enhanced heat shock responses [11]. Recently, HSP90B1 targeted therapies have been developed in cancer treatment including small molecular compounds [12]. However, a comprehensive understanding of HSP90B1’s role in cancer remains lacking. Hence, it is imperative to explore the regulatory functions and molecular mechanisms of HSP90B1, offering new perspectives and rationales for cancer diagnosis and therapy.

## Findings

### HSP90B1 expression analysis in pan-cancer and prognostic significance

**Fig. 1:**
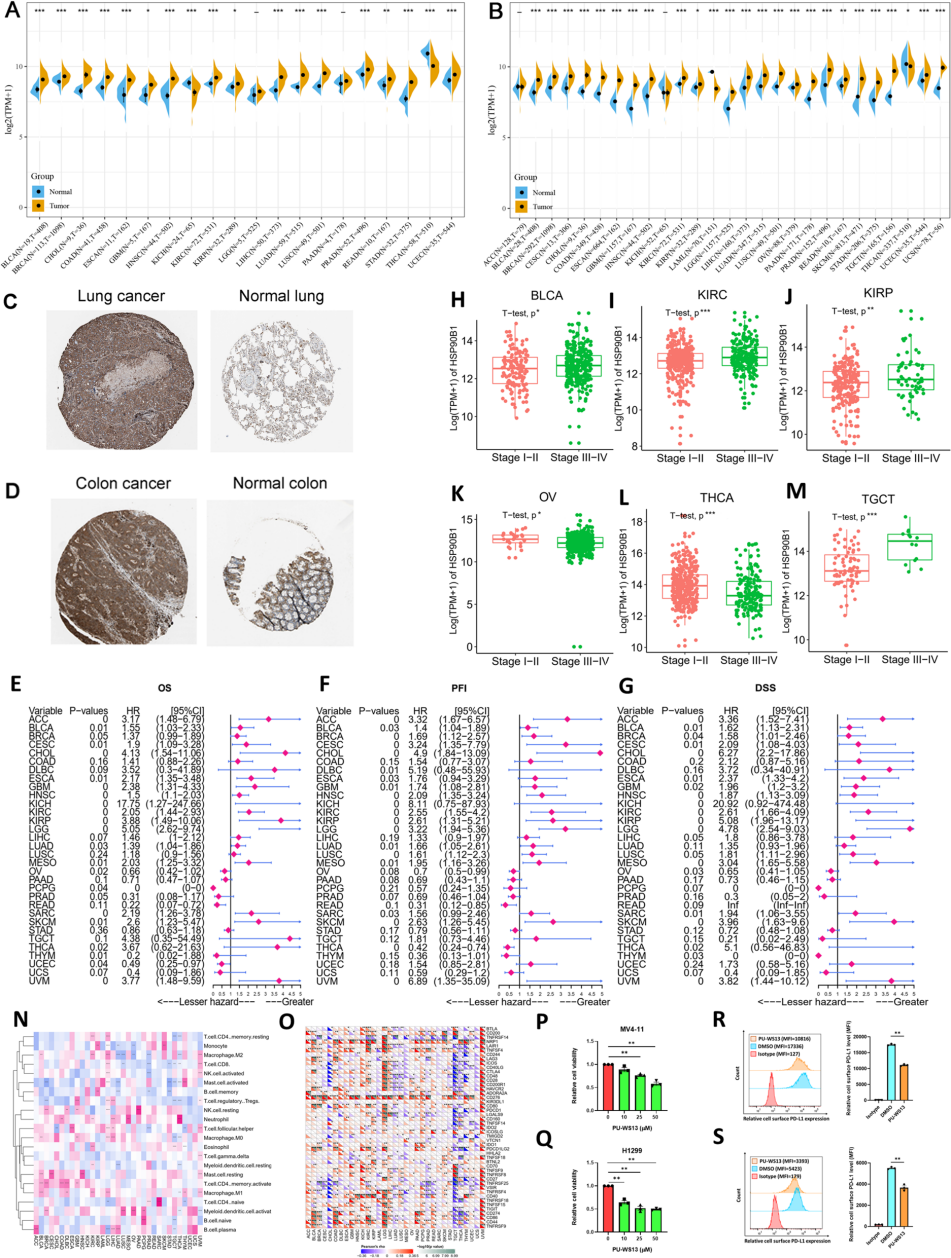
Analysis of variations in HSP90B1 expression and its prognostic significance across various cancer types in pan-cancer. **A** Differential expression of HSP90B1 between cancer and peritumoral tissue in multiple samples obtained from the TCGA database. Symbols “*”, “**”, and “***” denote statistical significance with *p* < 0.05, *p* < 0.01, and *p* < 0.001, respectively (Student t-test). **B** Differential expression of HSP90B1 between cancer tissue obtained from samples in the TCGA database and peritumoral tissue obtained from samples in the TCGA and GTEx database. Symbols “*”, “**”, and “***” denote statistical significance with *p* < 0.05, *p* < 0.01, and *p* < 0.001, respectively (Student t-test). **C**, **D** Protein expression profiling of HSP90B1 in lung cancer and adjacent normal lung tissue, as well as in colon cancer and normal colon tissue. **E**–**G** Forest plot of single Cox regression analysis depicting HRs of HSP90B1 in pan-cancer for OS, PFI and DSS. Only *p* < 0.05 signifies a significant association between HSP90B1 expression and cancer prognosis. HR > 1 suggests that elevated HSP90B1 expression is indicative of a heightened risk for poor prognosis. (H-M) Box plots depicting correlation between HSP90B1 expression and tumor staging in BLCA, KIRC, KIRP, OV, THCA and TGCT. The central box encapsulates the interquartile range (IQR) of HSP90B1 expression, featuring the median as a line within the box. The whiskers extend to values within 1.5 times the IQR, representing the range of maximum and minimum expression. Symbols “*”, “**”, and “***” denote statistical significance with *p* < 0.05, *p* < 0.01, and *p* < 0.001, respectively (Student t-test). (N–O) Heat maps demonstrating correlation between HSP90B1 expression and immune infiltrating cells, immune checkpoint genes in Pan-Cancer. Symbols “*”, “**”, and “***” denote statistical significance with *p* < 0.05, *p* < 0.01, and *p* < 0.001, respectively (Pearson correlation).(P-Q) Effect of HSP90B1 inhibitor PU-WS13 on proliferation of leukemia cells (MV4-11, 48 h treatment, *n* = 3) and solid tumor cells (lung cancer H1299, 48 h treatment, *n* = 3).Symbols “*” and “**”denote statistical significance with *p* < 0.05 and *p* < 0.01 respectively (Student t-test).(R-S) Effect of HSP90B1 inhibitor PU-WS13 on the immune checkpoint PD-L1 cell surface expression level in leukemic cells (MV4-11, 75 μM 48 h treatment) and solid tumor cells (lung cancer H1299, 50 μM 48 h treatment). Symbol “**” denotes statistical significance with *p* < 0.01 (Student t-test)

To investigate the relationship between HSP90B1 and various cancers, we assessed differences in HSP90B1 expression across different cancer and normal tissues. The results demonstrated that HSP90B1 expression in cancer tissues, including BLCA, BRCA, CHOL, COAD, ESCA, HNSC, etc., were significantly higher than those in normal tissues according to the TCGA database (all *p* < 0.05) (Fig. 1A; Suppl. Table S1). In addition, considering the limited number of samples for peritumoral tissue in the TCGA database alone, we conducted a combined analysis of normal samples from both the TCGA and GETx databases, yielding consistent results (all *p* < 0.05) (Fig. 1B). Moreover, HSP90B1expression was higher in colon and lung cancer tissues in comparison with their respective normal tissues through analysis of Human Protein Atlas (HPA) database (Fig. 1C, D).

In the next place, we performed Kaplan–Meier overall survival (OS) analysis using the TCGA database. Our findings indicated that HSP90B1 serves as a risk factor for patients with ACC, CHOL, BLCA, CESC, GBM, KICH, etc., while acting as a protective factor for patients with LAML, OV, PCPG, THYM, UCEC (all *p* < 0.05) (Suppl. Fig. S1). Similar trends were observed in disease-specific survival (DSS) and progression-free interval (PFI) results, reinforcing HSP90B1 as a risk factor (Suppl. Figs. S2, S3). Subsequently, we performed a univariate COX analysis, unveiling that HSP90B1 stands as a risk factor for patients with ACC, BLCA, CESC, CHOL, ESCA, GBM, etc., while offering a protective effect for patients with LAML, OV, PCPG, UCEC (Fig. 1E). Consistent results were observed in DSS and PFI analyses (Fig. 1F, G). Furthermore, when stratifying tumor patients into stages I-II and III-IV based on disease progression, high expression levels of HSP90B1 were significantly increased in advanced stages for most cancers, including BLCA, KIRC, KIRP, and TGCT (all *p* < 0.05) (Fig. 1H, I, J and M), and even in early stages for certain cancer types like OV and THCA (Fig. 1K, L). Additionally, MethSurv [13] analysis revealed that methylation of cg21049487 and cg19615102 in the HSP90B1 gene served as a protective factor across BRCA, CESC, ESCA, GBM, KIRP, and LIHC (Suppl. Fig. S4A). Specifically, high methylation of cg21049487 in HSP90B1 was associated with a better prognosis in GBM, BRCA, and CESC (Suppl. Fig. S4B-D). Similarly, elevated methylation of cg19615102 in HSP90B1 was correlated with a favorable prognosis in ESCA (Suppl. Fig. S4E).

Furthermore, we investigated the correlation of HSP90B1 expression in 33 tumors with infiltrating immune cells within the tumor microenvironment. HSP90B1 exhibited a positive correlation with the extent of neutrophil infiltration in the majority of tumors. Particularly, for M0 macrophages, their infiltration levels were positively correlated with HSP90B1 expression in BLCA, GBM, LGG, LIHC, and negatively correlated with TGCT, THCA (all *P* < 0.05). In addition, in HCA, HSP90B1 expression was negatively correlated with the infiltration level of M1 macrophages (all *p* < 0.05). Conversely, HSP90B1 expression in M2 macrophages exhibited a negative correlation with KIRC, LGG, UVM (all *p* < 0.05). At last, HSP90B1 expression demonstrated a negative correlation with the infiltrating CD8 + T cells in LUSC, PRAD, STAD, TGCT, THCA. (all *p* < 0.05) (Fig. 1N).

Subsequently, extensive studies have demonstrated the pivotal role of immune checkpoint (ICP) genes in influencing both the efficacy of immunotherapy and the behavior of immune cells. Our results revealed that in the great majority of tumors, the expression of HSP90B1 showed a positive correlation with the expression of NRP1, CD276, CD44, CD274 (PD-L1) and, conversely, a negative correlation with the expression of TNFRSF25. It is noteworthy that high expression of these genes tends to have a worse prognosis, the positive association between HSP90B1 expression with these immune checkpoint genes may also symbolize a poorer prognosis (Fig. 1O).

In addition, we assessed the impact of the HSP90B1 inhibitor on leukemic and solid tumor cell models. The well-known developed HSP90B1 targeted chemical inhibitor PU-WS13 was involved in these validation assays. Remarkably, the treatment with the HSP90B1 inhibitor resulted in a significant reduction in cancer cell proliferation capabilities (Fig. 1P-Q; Suppl. Figure S5A-C), coupled with a substantial decrease in the levels of cell surface PD-L1 (Fig. 1R-S). Besides, we also performed RT-qPCR assay to validate that flt3-itd mutation burden MV4-11 cells displayed higher HSP90B1 level than RS4;11 cells with wild type flt3 (Suppl. Fig. S5D). Furthermore, in order to seek for potential anti-tumor drugs targeting HSP90B1-associated genes, we conducted analysis from the Connectivity Map (CMap) database and identified 57 HSP inhibitors [14], which demonstrated proliferative-suppressive effects on cancer cell lines such as SKB, A375, PC3, and SW480 (connectivity score < 0) (Suppl. Table S2).In summary, the data presented above suggested that HSP90B1 holds promise as a potential cancer biomarker and may exert a facilitative role in the malignancy development of multiple cancers.

### Correlation analyses between HSP90B1 expression and cancer hallmarks provide a therapeutic window

**Fig. 2:**
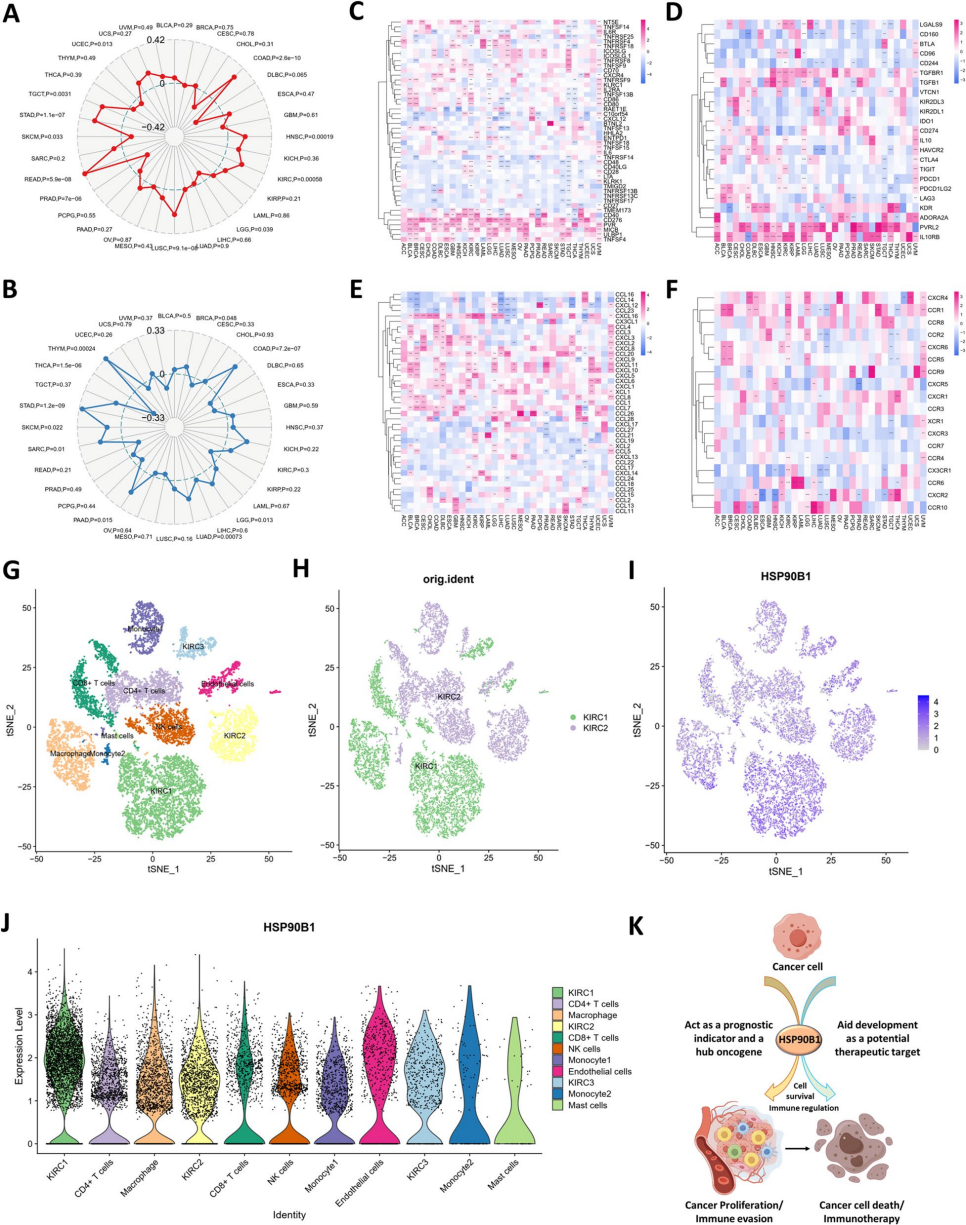
Correlations between HSP90B1 expression and genetic alterations, immune-related genes and TME. **A**, **B** Radar charts illustrating relationship between HSP90B1 expression and MSI, TMB in pan-cancer. There are radial axes emanating from a common center, point in each axis corresponds to correlation coefficient(Spearman correlation). **C**-**F** Heat maps of association between HSP90B1 expression with the immune stimulating genes, immunosuppressive genes, chemokines and chemokine receptors. Symbols “*”, “**”, and “***” denote statistical significance with *p* < 0.05, *p* < 0.01, and *p* < 0.001, respectively (Pearson correlation). **G**, **H** tSEN plot revealing the landscape of distinct cell types within the single-cell transcriptomic atlas, distinct clusters and HSP90B1 expression in KIRC. **J** Comparison of HSP90B1 expression across diverse tumor-infiltrating lymphocyte within the TME of KIRC. **K** A conceptual framework outlining the role of HSP90B1 in both cancer development and therapeutic interventions

Subsequently, we analyzed the following relationships between HSP90B1 expression and tumor mutational burden (TMB) in various tumors, revealing a positive correlation between HSP90B1 expression and TMB (Fig. 2A). We also analyzed the correlation between the gene expression of HSP90B1 and microsatellite instability(MSI), and the results indicated in most tumors, the expression of HSP90B1 was positively correlated with MSI (Fig. 2B). We further delved into the genetic alterations of HSP90B1 within various tumors utilizing data from the TCGA cohort. Notably, the frequency of HSP90B1 alterations was most pronounced in UCEC patients (> 6%) and was associated with "mutation" in most cancers (Suppl. Fig. S6A). Concurrently, an assessment of the correlation between HSP90B1 expression and gene copy number variation (CNV) in different tumor types revealed a consistent positive correlation between CNV and HSP90B1 expression(Suppl. Fig. S6B). Further insight was gained through the heat map illustrating the correlation between gene mutations and HSP90B1 expression, notably, mutations in TP53, MET, and MTOR exhibited positive correlations with HSP90B1 expression (Suppl. Fig. S6C).

The proportion of immune cells and stromal cells within a tumor has a significant effect on the prognosis. Through a comprehensive analysis of Tumor ImmuneScore, StromalScore, and ESTIMATEScore (Supplementary Figs. S7, S8 and S9), we observed a negative correlation between HSP90B1 expression and tumor purity in BLCA, KIRC, and PCPG (all *p* < 0.05). Conversely, in LUAD, PRAD, STAD, THCA, and UCEC, HSP90B1 expression exhibited a positive correlation with tumor purity (all *p* < 0.05). Utilizing Gene Set Enrichment Analysis (GSEA), we delineated the functional enrichment of genes in cohorts with high and low HSP90B1 expression. The high expression of HSP90B1 was associated with propanoate metabolism, cell cycle and lysine degradation. Conversely, the low expression of HSP90B1 was associated with autoimmune thyroid disease, asthma, allograft rejection and intestinal immune network for IgA production (Suppl. Fig. S10). These findings underscored the close relationship of HSP90B1 with the tumor immune microenvironment, cellular metabolism, and cell cycle regulation.

Additionally, we also investigated the correlation between HSB90B1 and immunosuppressive genes. The analysis of 46 immunostimulatory genes in pan-cancer showed that HSP90B1 expression was positively correlated with CD40, CD270, PVR, MICB, ULBP1, TNFSF4, while exhibiting a negative correlation with CD48 and CD40LG in most cancers (Fig. 2C). Moreover, HSP90B1 expression demonstrated positive correlations with PVRL2, IL10RB, TGFBR1, and TGFB1 within a subset of 20 immunosuppressive genes (Fig. 2D). What’s more, we also discovered the close correlation of HSP90B1 expression with chemokines and their receptor genes (Fig. 2E, F). Taken together, HSP90B1 expression was mainly positively correlated with immunostimulatory genes and mainly negatively correlated with immunosuppressive genes.

Moreover, single-cell transcriptional analysis was performed on 2 KIRC samples. Employing the tSNE algorithm for cell clustering analysis, we classified the cells into 11 distinct clusters, namely KIRC1, KIRC2, KIRC3, monocyte1, monocyte2, macrophage, mast cells, endothelial cells, NK cells, CD4 + T cells, and CD8 + T cells (Fig. 2G). Intriguingly, it was observed that tumor cells from disparate sources of KIRC samples shared a common cluster (KIRC3) and unique clusters (KIRC1 and KIRC2) (Fig. 2H), underscoring the heterogeneity of KIRC cell types. In the next place, we explored the HSP90B1 expression of infiltrating immune cells within the tumor microenvironment in KIRC and conducted a comparative analysis of HSP90B1 expression across the identified cell types (Fig. 2I). Interestingly, a significant difference in HSP90B1 expression was observed among the 11 cell types (Fig. 2J). Moreover, HSP90B1 expression was highest in KIRC cells, but lowest in mast cells. The expression of HSP90B1 was markedly higher in tumor cells than in immune cells (*p* < 0.05). Therefore, targeting HSP90B1 may be more lethal to tumor cells, which provides a strategic therapeutic window.

## Conclusion

In summary, we performed a comprehensive evaluation of HSP90B1 in cancer, which revealed its underlying role as a prognostic indicator for patients and its role in the regulation of tumor development, metabolism and immune microenvironment (Fig. 2K). HSP90B1 has pro-cancer effects and is closely related to tumor development and immunity invasion, and therefore, may become a new biomarker of cancer diagnosis, prognosis and a novel target for future cancer therapy.

### Supplementary Information

**Additional file 1:** **Supplement Figure S1**. Kaplan-Meier survival curves for OS in pan-cancer stratified by expression of HSP90B1. *p* < 0.05 signifies a significant association between HSP90B1 expression and cancer prognosis. HR > 1 suggests that elevated HSP90B1 expression is indicative of a heightened risk for poor prognosis.

**Additional file 2:** **Supplement Figure S2.** Kaplan-Meier survival curves for DSS in pan-cancer stratified by expression of HSP90B1. *p* < 0.05 signifies a significant association between HSP90B1 expression and cancer prognosis. HR > 1 suggests that elevated HSP90B1 expression is indicative of a heightened risk for poor prognosis.

**Additional file 3:** **Supplement Figure S3.** Kaplan-Meier survival curves for PFI in patients with cancer with high versus low levels of HSP90B1 expression. *p* < 0.05 signifies a significant association between HSP90B1 expression and cancer prognosis. HR > 1 suggests that elevated HSP90B1 expression is indicative of a heightened risk for poor prognosis.

**Additional file 4:** **Supplement Figure S4.** Prognostic analysis of HSP90B1 methylation in pan-cancer. (A) Forest plot assessing the effect of HSP90B1 methylation on cancer prognosis. HR < 1 suggests that HSP90B1 methylation is indicative of a reduced risk for poor prognosis. (B-D) Kaplan-Meier survival curves for OS in GBM, BRCA and CESC stratified by methylation level of cg21049487 in HSP90B1. *p* < 0.05 signifies a significant association between methylation level of cg21049487 in HSP90B1 and cancer prognosis. HR < 1 suggests that elevated methylation level of cg21049487 in HSP90B1 is indicative of a reduced risk for poor prognosis. (E) Kaplan-Meier survival curves for OS in ESCA stratified by methylation level of cg19615102 in HSP90B1. *p* < 0.05 signifies a significant association between methylation level of cg19615102 and cancer prognosis. HR < 1 suggests that elevated methylation level of cg19615102 is indicative of a reduced risk for poor prognosis.

**Additional file 5:** **Supplement Figure S5.** HSP90B1 targeted chemical inhibitor PU-WS13 significantly inhibited cancer cell proliferation and oncogene probably induced higher HSP90B1 level. (A-C) Effect of HSP90B1 inhibitor PU-WS13 on proliferation of leukemia cells (Molm13, 48h treatment, *n*=3) and solid tumor cells (colorectal cancer HCT116, 48h treatment, *n*=3; lung cancer H1975, 48h treatment, *n*=3;).Symbols “*” and “**” denote statistical significance with *p* < 0.05 and *p* < 0.01 respectively (Student t-test). (D) RT-qPCR experiments to validate the HSP90B1 levels using two comparable cancer cell lines of RS4;11 (acute lymphoblastic leukemia cell line with wild type flt3) and MV4-11 (acute myeloid leukemia cell line with flt3-itd mutation). Symbol “**” denotes statistical significance with *p* < 0.01 respectively (Student t-test)

**Additional file 6:** **Supplement Figure S6.** HSP90B1 and tumor gene variation analysis. (A) Histogram of the proportion of different mutation modes of HSP90B1 gene in pan-cancer. (B) Lollipop chart depicting the correlation between HSP90B1 expression and CNV in pan-cancer. (C) Heat map exhibiting the correlation between HSP90B1 expression and the specific gene mutation. Symbols “*”, “**”, and “***” denote statistical significance with *p* < 0.05, *p* < 0.01, and *p* < 0.001, respectively (Pearson correlation).

**Additional file 7:** **Supplement Figure S7.** Scatter plot of correlation between HSP90B1 expression and ImmuneScore in pan-cancer by ESTIMATE bioinformatics tool.

**Additional file 8:** **Supplement Figure S8.** Scatter plot of correlation between HSP90B1 expression and StromalScore in pan-cancer by ESTIMATE bioinformatics tool.

**Additional file 9:** **Supplement Figure S9.** Scatter plot of correlation between HSP90B1 expression and ESTIMATEScore in Pan-Cancer by ESTIMATE bioinformatics tool.

**Additional 10:** **Supplement Figure S10.** Exploration of signaling pathways associated with HSP90B1 expression. (A-B) Enrichment analysis in the KEGG pathway for HSP90B1 high-expression group and HSP90B1 low-expression group. (C-D) Enrichment analysis in the HALLMARK pathway for HSP90B1 high-expression group and HSP90B1 low-expression group.

**Additional 11:** **Supplementary Table S1.** Full names and abbreviations of the tumor types involved in this study.

**Additional 12:** **Supplementary Table S2.** Analysis of potential anti-tumor drugs targeting HSP90B1-associated genes based on the CMap database.Each positive connectivity score signifies a positive correlation between the drug perturbation expression profile and the disease perturbation expression profile, which implies that the agent may induce or exacerbate the associated disease state. Conversely, each negative connectivity score indicates a negative correlation between the drug perturbation expression profile and the disease perturbation expression profile, suggesting that the agent may alleviate or even reverse the associated disease state.

## Data Availability

The dataset supporting the conclusions of this article is included within the article. The data supporting the findings of this study are deposited in the TCGA,GEO, Methsurv and cMAP databases. The single-cell sequencing datasets can be found in the online repositories of GEO (GSE152938).
