# Supplementary figures and images for: Identification of HSP90B1 in pan-cancer hallmarks to aid development of a potential therapeutic target
Source: Mol Cancer. 2024 Jan 20;23:19. doi: 10.1186/s12943-023-01920-w (PMC10799368; doi:10.1186/s12943-023-01920-w)

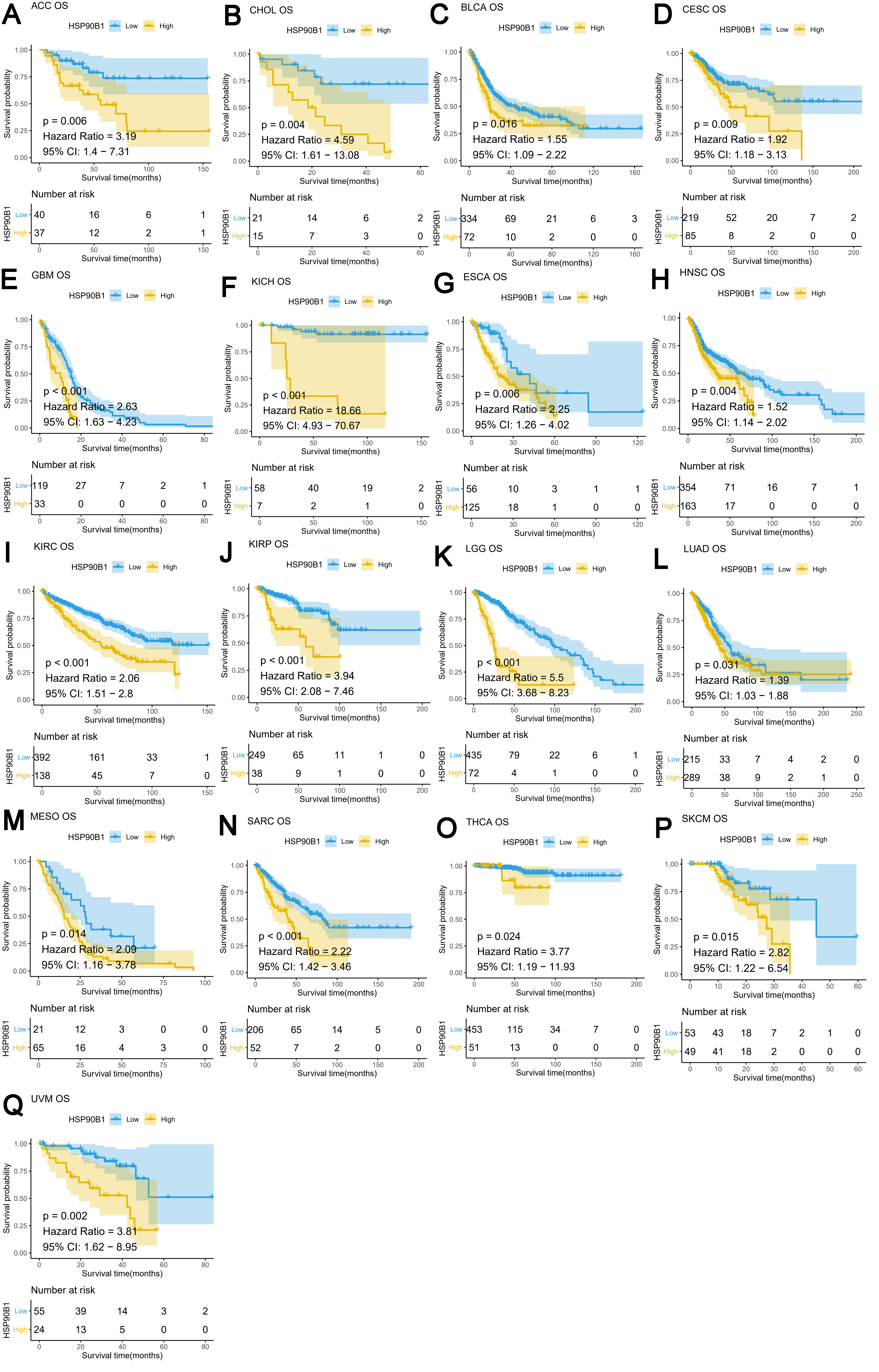

Supplement: Supplementary file 1 — Additional file 1: Supplement Figure S1. Kaplan-Meier survival curves for OS in pan-cancer stratified by expression of HSP90B1. p < 0.05 signifies a significant association between HSP90B1 expression and cancer prognosis. HR > 1 suggests that elevated HSP90B1 expression is indicative of a heightened risk for poor prognosis. [file 12943_2023_1920_MOESM1_ESM.tif]

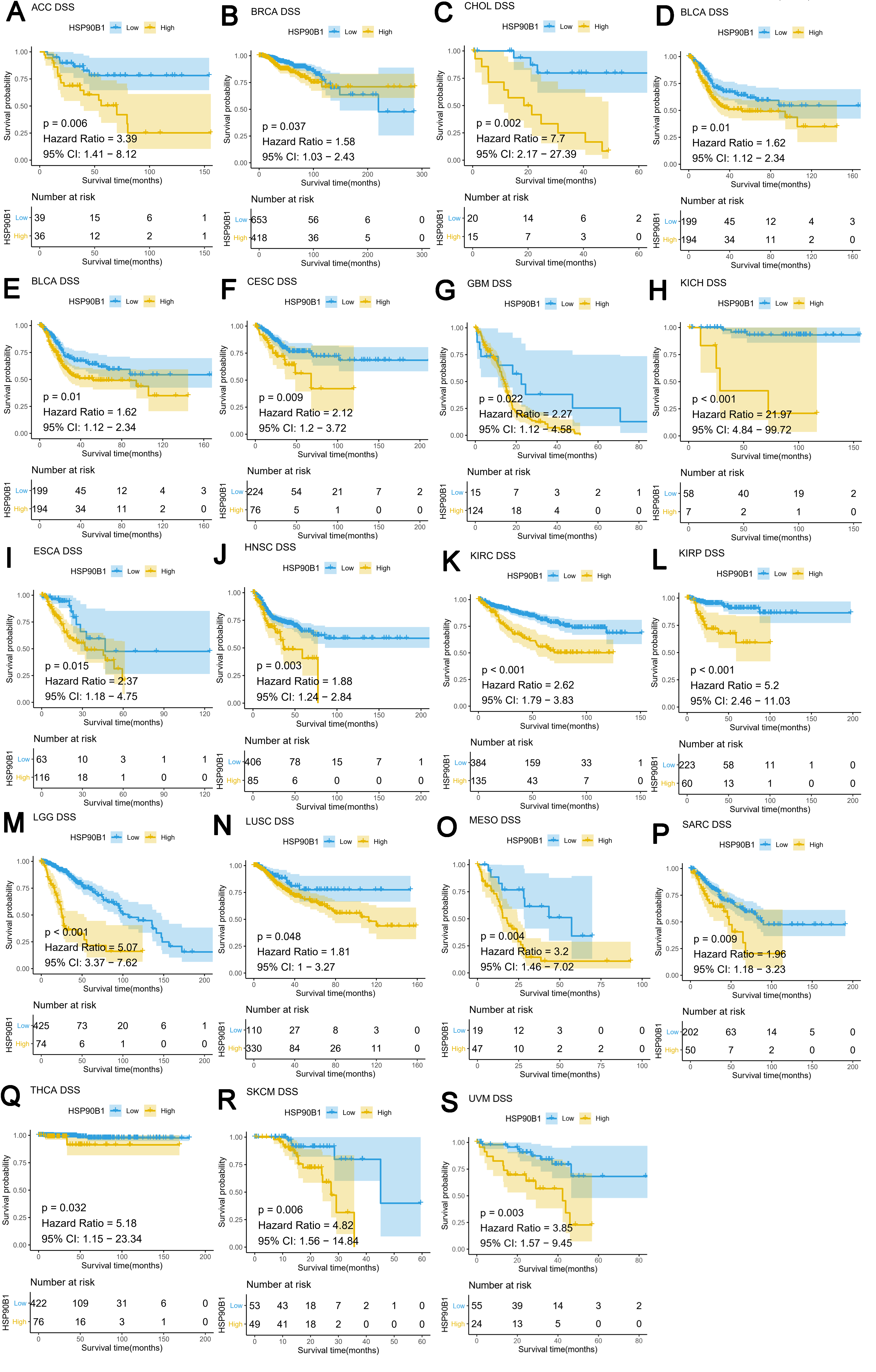

Supplement: Supplementary file 2 — Additional file 2: Supplement Figure S2. Kaplan-Meier survival curves for DSS in pan-cancer stratified by expression of HSP90B1. p < 0.05 signifies a significant association between HSP90B1 expression and cancer prognosis. HR > 1 suggests that elevated HSP90B1 expression is indicative of a heightened risk for poor prognosis. [file 12943_2023_1920_MOESM2_ESM.tif]

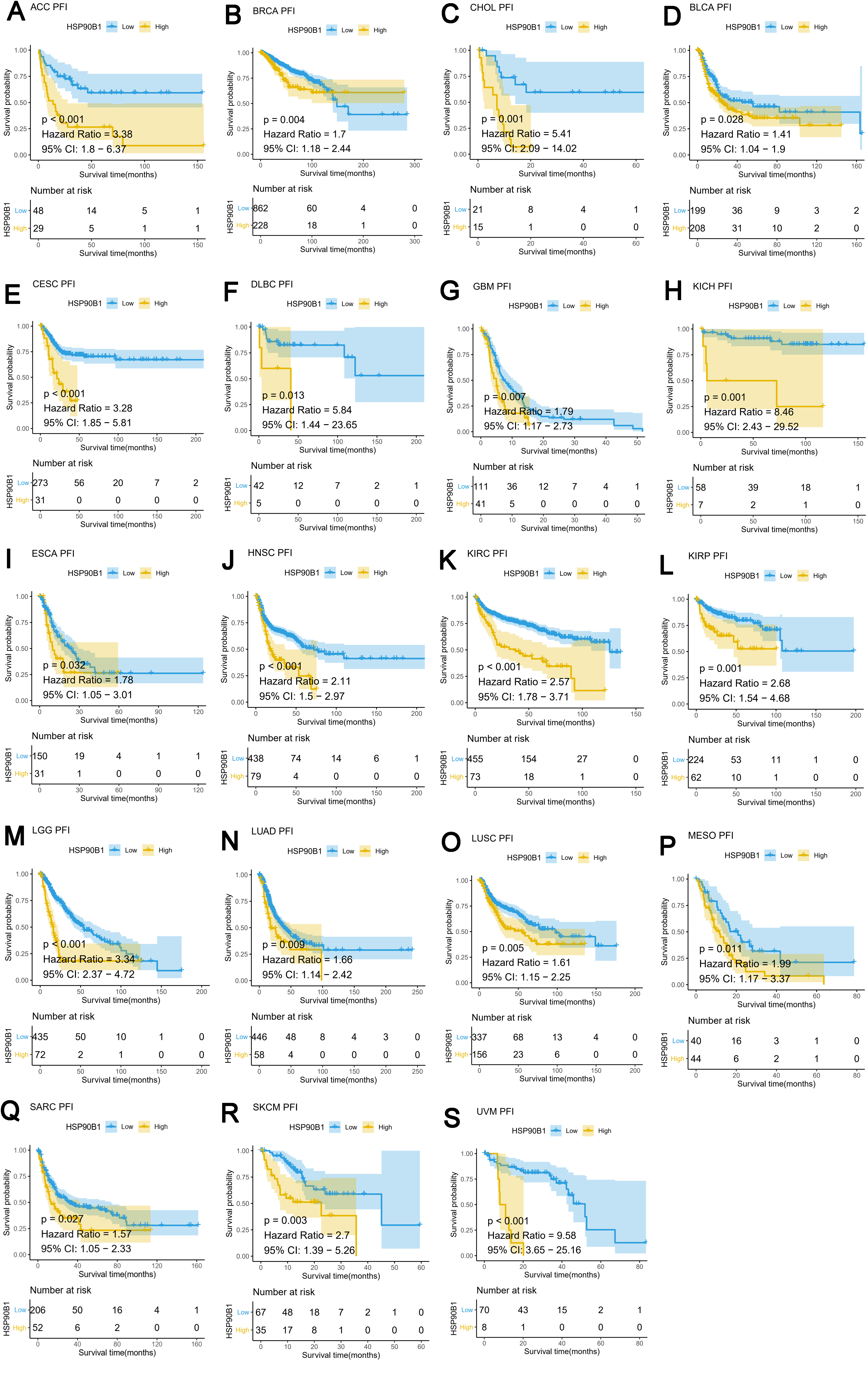

Supplement: Supplementary file 3 — Additional file 3: Supplement Figure S3. Kaplan-Meier survival curves for PFI in patients with cancer with high versus low levels of HSP90B1 expression. p < 0.05 signifies a significant association between HSP90B1 expression and cancer prognosis. HR > 1 suggests that elevated HSP90B1 expression is indicative of a heightened risk for poor prognosis. [file 12943_2023_1920_MOESM3_ESM.tif]

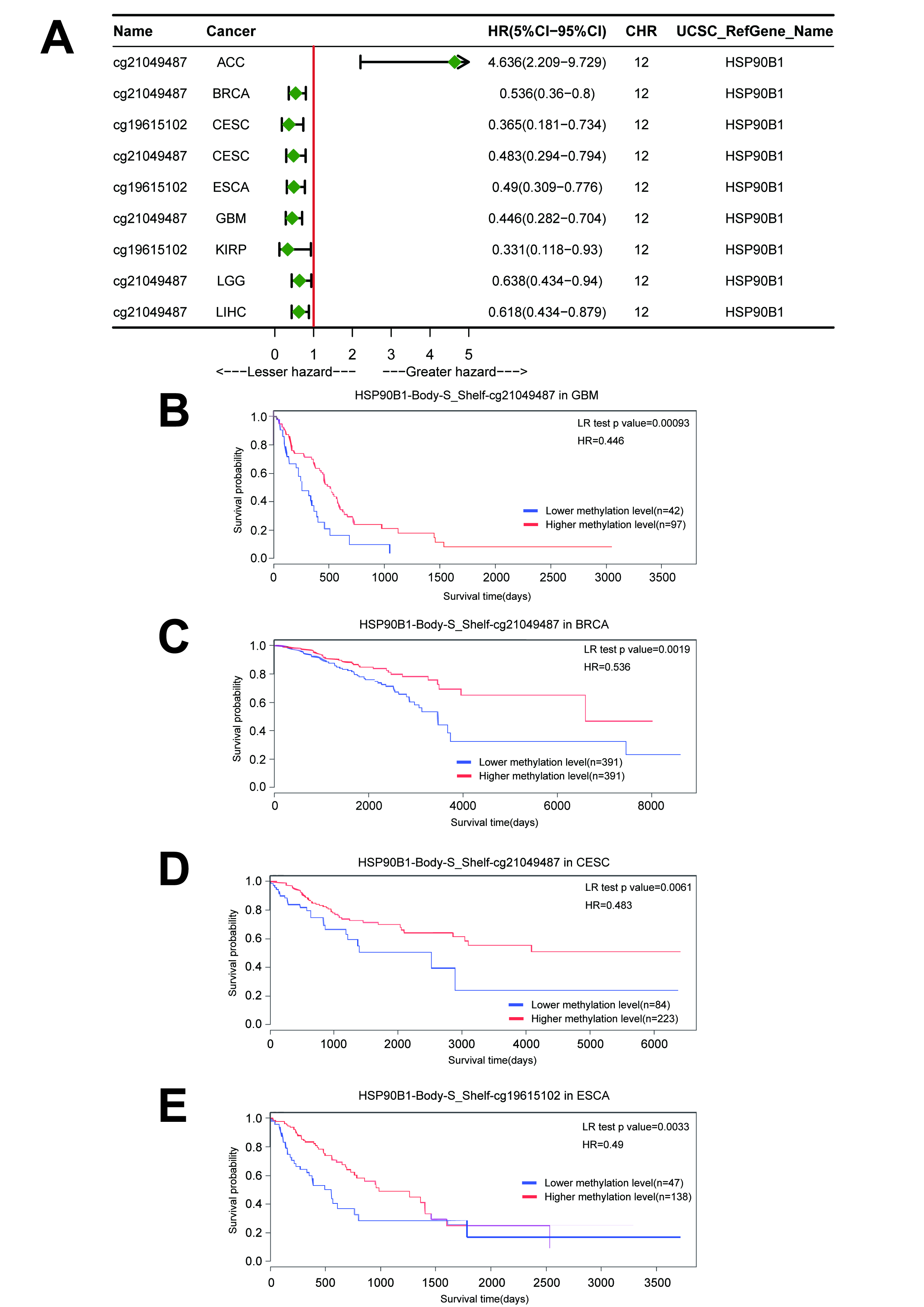

Supplement: Supplementary file 4 — Additional file 4: Supplement Figure S4. Prognostic analysis of HSP90B1 methylation in pan-cancer. (A) Forest plot assessing the effect of HSP90B1 methylation on cancer prognosis. HR < 1 suggests that HSP90B1 methylation is indicative of a reduced risk for poor prognosis. (B-D) Kaplan-Meier survival curves for OS in GBM, BRCA and CESC stratified by methylation level of cg21049487 in HSP90B1. p < 0.05 signifies a significant association between methylation level of cg21049487 in HSP90B1 and cancer prognosis. HR < 1 suggests that elevated methylation level of cg21049487 in HSP90B1 is indicative of a reduced risk for poor prognosis. (E) Kaplan-Meier survival curves for OS in ESCA stratified by methylation level of cg19615102 in HSP90B1. p < 0.05 signifies a significant association between methylation level of cg19615102 and cancer prognosis. HR < 1 suggests that elevated methylation level of cg19615102 is indicative of a reduced risk for poor prognosis. [file 12943_2023_1920_MOESM4_ESM.tif]

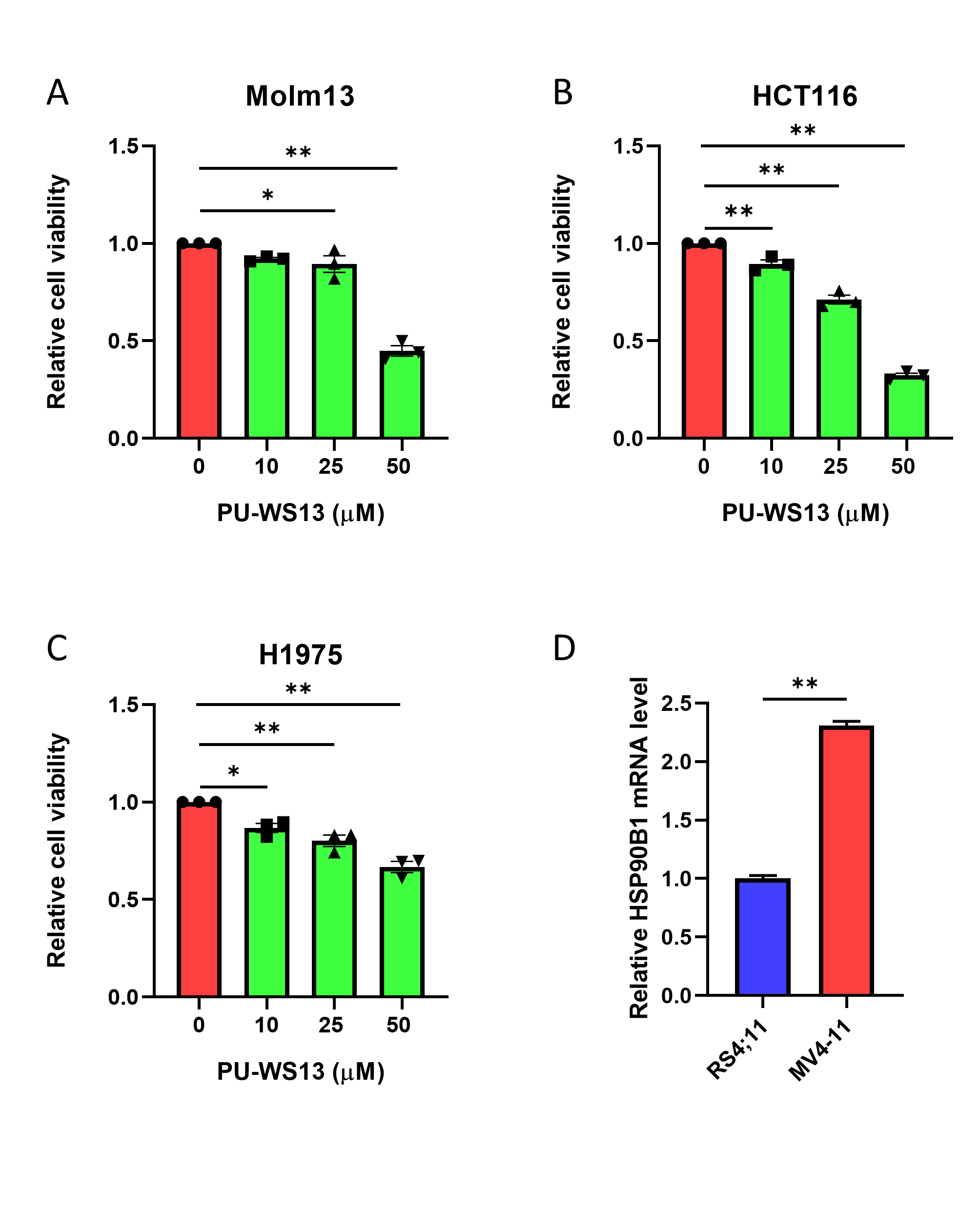

Supplement: Supplementary file 5 — Additional file 5: Supplement Figure S5. HSP90B1 targeted chemical inhibitor PU-WS13 significantly inhibited cancer cell proliferation and oncogene probably induced higher HSP90B1 level. (A-C) Effect of HSP90B1 inhibitor PU-WS13 on proliferation of leukemia cells (Molm13, 48h treatment, n=3) and solid tumor cells (colorectal cancer HCT116, 48h treatment, n=3; lung cancer H1975, 48h treatment, n=3;).Symbols “*” and “**” denote statistical significance with p < 0.05 and p < 0.01 respectively (Student t-test). (D) RT-qPCR experiments to validate the HSP90B1 levels using two comparable cancer cell lines of RS4;11 (acute lymphoblastic leukemia cell line with wild type flt3) and MV4-11 (acute myeloid leukemia cell line with flt3-itd mutation). Symbol “**” denotes statistical significance with p < 0.01 respectively (Student t-test) [file 12943_2023_1920_MOESM5_ESM.tif]

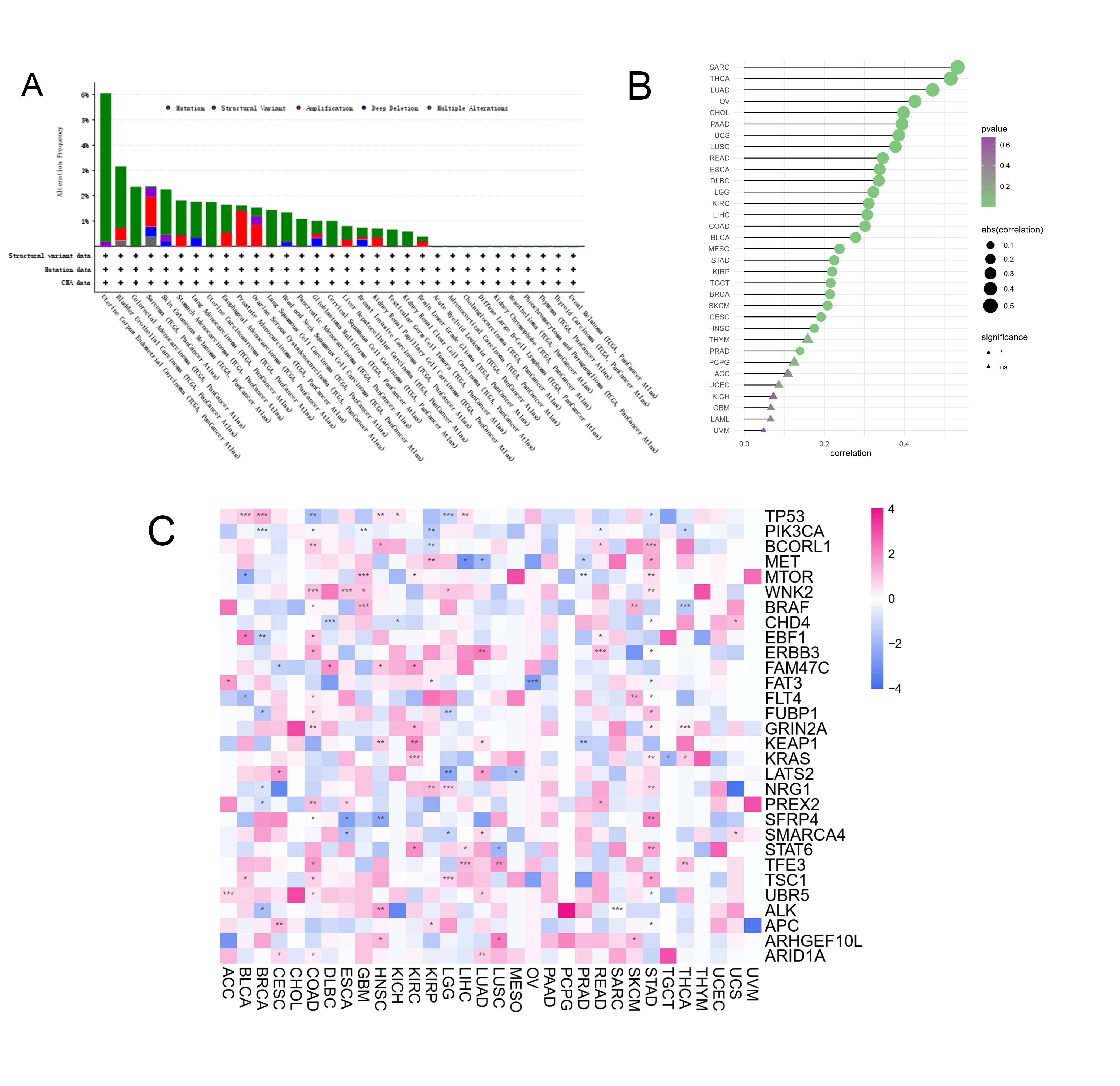

Supplement: Supplementary file 6 — Additional file 6: Supplement Figure S6. HSP90B1 and tumor gene variation analysis. (A) Histogram of the proportion of different mutation modes of HSP90B1 gene in pan-cancer. (B) Lollipop chart depicting the correlation between HSP90B1 expression and CNV in pan-cancer. (C) Heat map exhibiting the correlation between HSP90B1 expression and the specific gene mutation. Symbols “*”, “**”, and “***” denote statistical significance with p < 0.05, p < 0.01, and p < 0.001, respectively (Pearson correlation). [file 12943_2023_1920_MOESM6_ESM.tif]

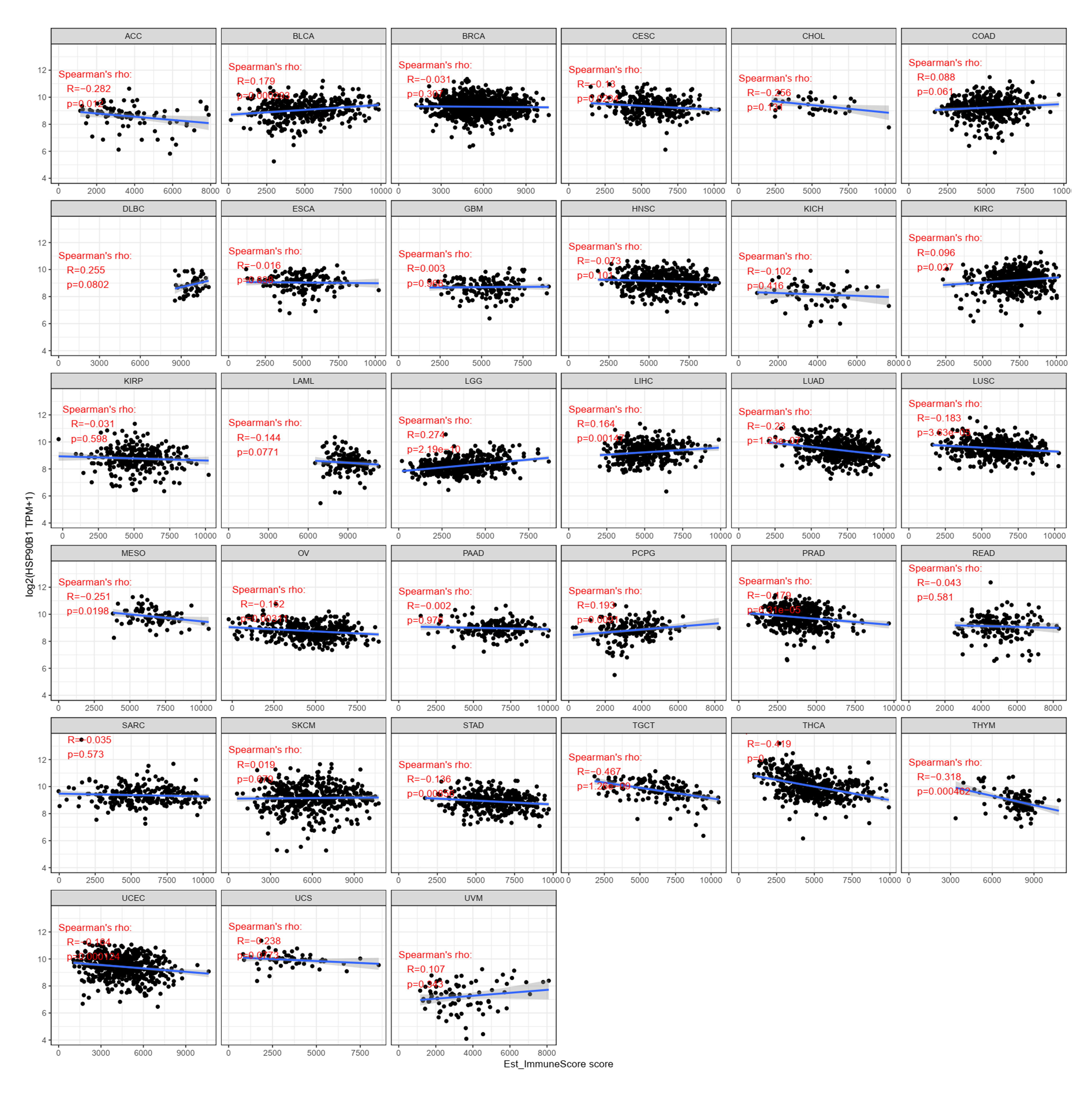

Supplement: Supplementary file 7 — Additional file 7: Supplement Figure S7. Scatter plot of correlation between HSP90B1 expression and ImmuneScore in pan-cancer by ESTIMATE bioinformatics tool. [file 12943_2023_1920_MOESM7_ESM.tif]

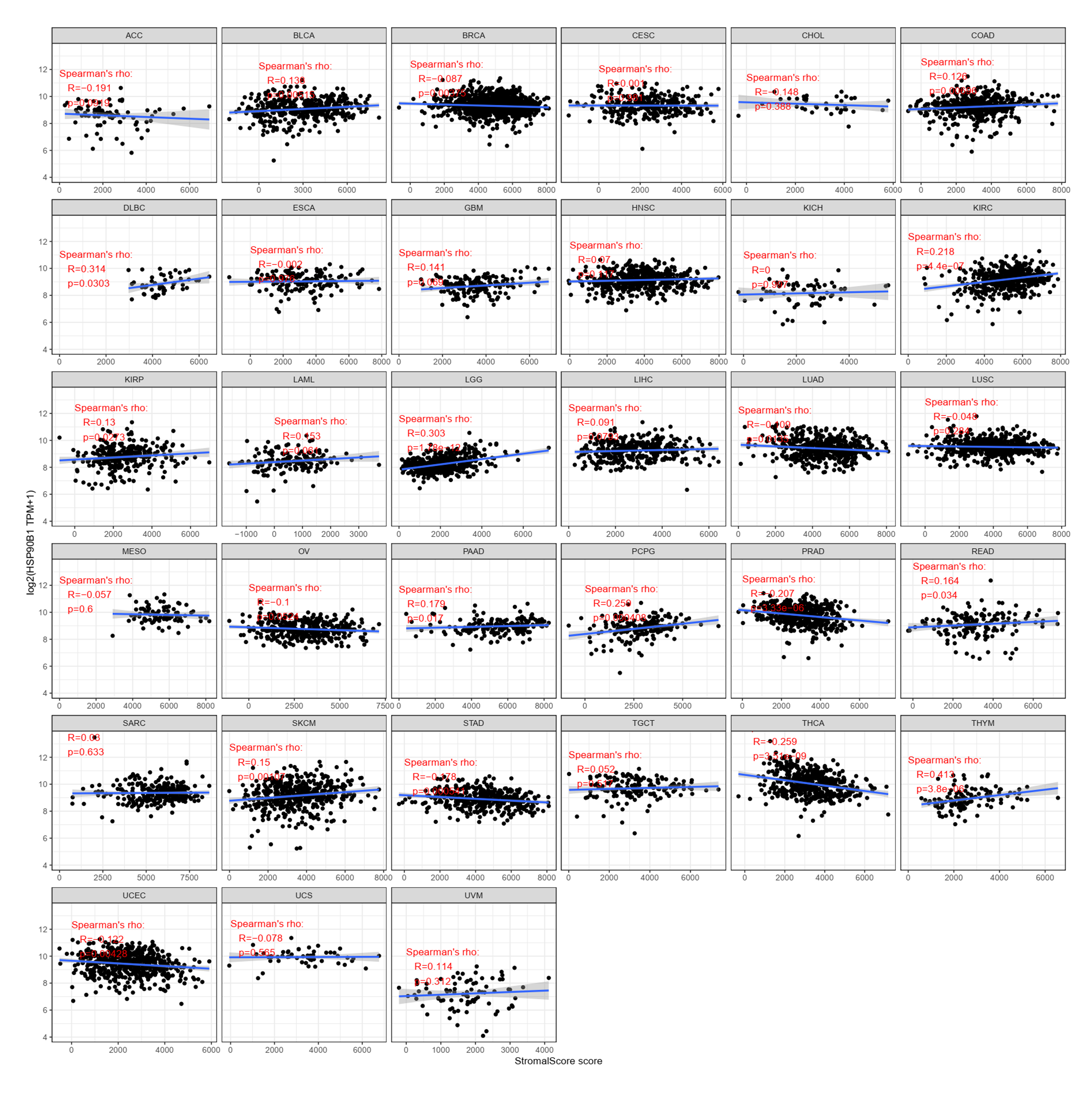

Supplement: Supplementary file 8 — Additional file 8: Supplement Figure S8. Scatter plot of correlation between HSP90B1 expression and StromalScore in pan-cancer by ESTIMATE bioinformatics tool. [file 12943_2023_1920_MOESM8_ESM.tif]

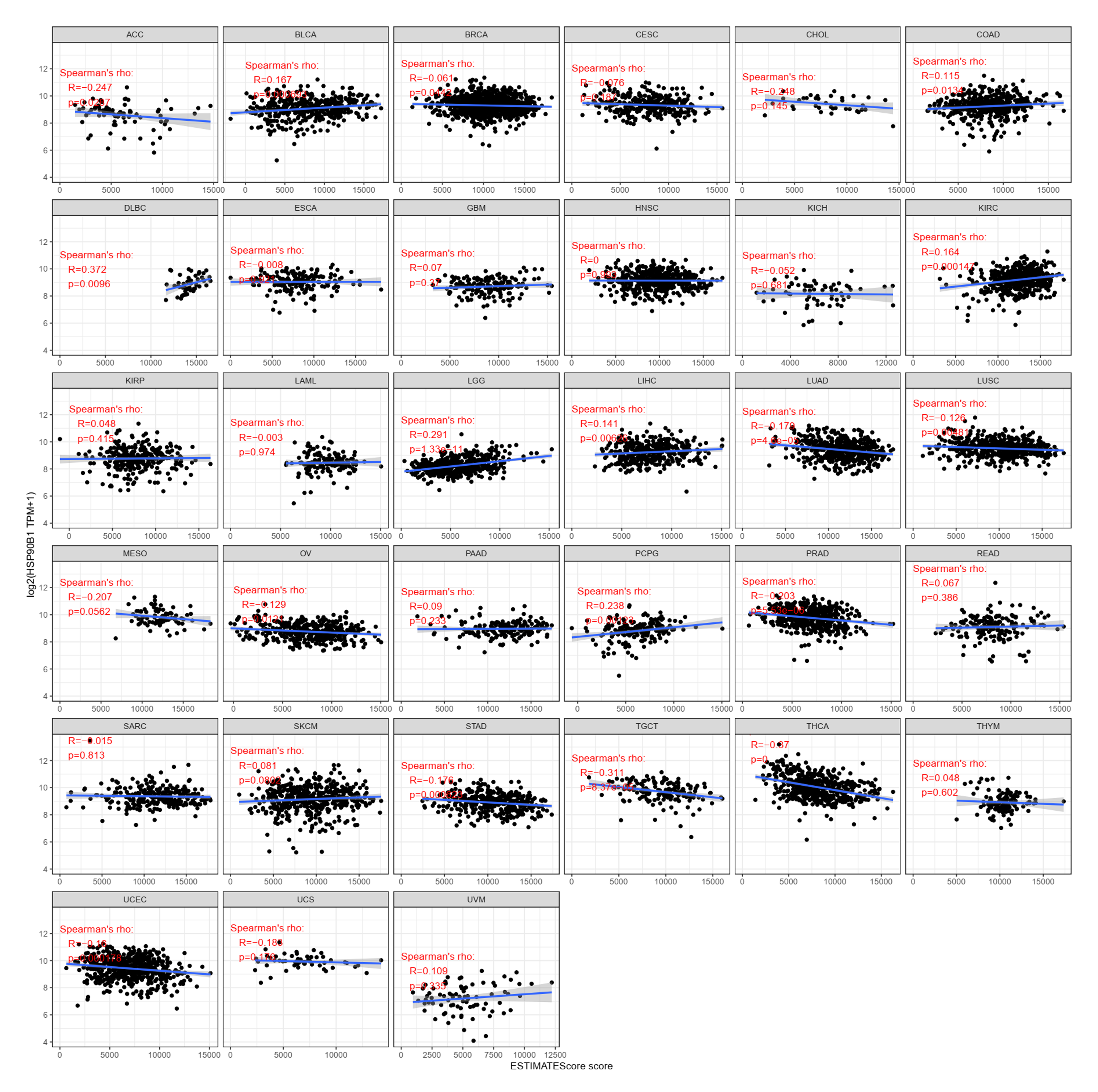

Supplement: Supplementary file 9 — Additional file 9: Supplement Figure S9. Scatter plot of correlation between HSP90B1 expression and ESTIMATEScore in Pan-Cancer by ESTIMATE bioinformatics tool. [file 12943_2023_1920_MOESM9_ESM.tif]

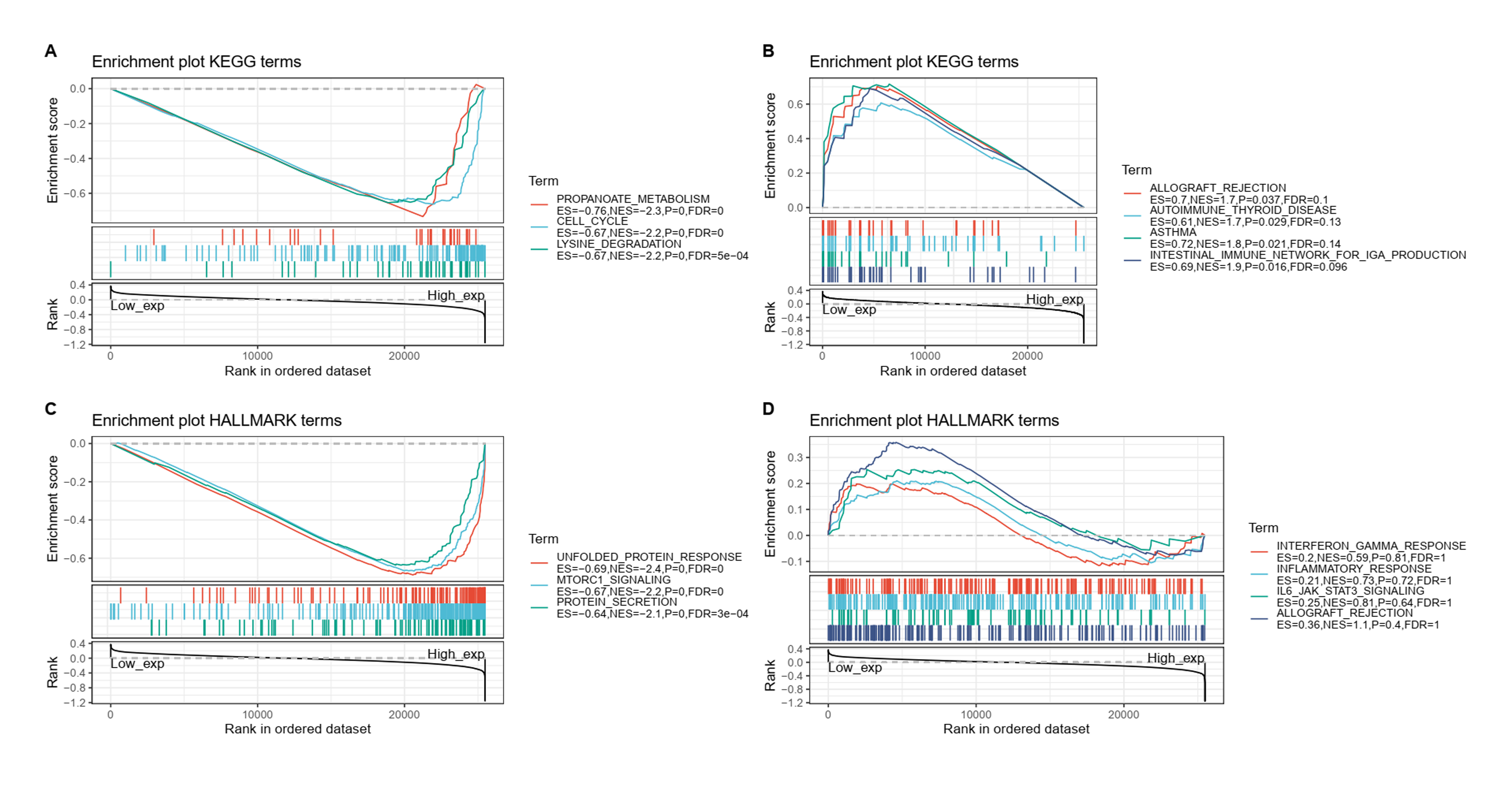

Supplement: Supplementary file 10 — Additional 10: Supplement Figure S10. Exploration of signaling pathways associated with HSP90B1 expression. (A-B) Enrichment analysis in the KEGG pathway for HSP90B1 high-expression group and HSP90B1 low-expression group. (C-D) Enrichment analysis in the HALLMARK pathway for HSP90B1 high-expression group and HSP90B1 low-expression group. [file 12943_2023_1920_MOESM10_ESM.tif]
