# Supplementary material for: Identification of HSP90B1 in pan-cancer hallmarks to aid development of a potential therapeutic target
Source: Mol Cancer. 2024 Jan 20;23:19. doi: 10.1186/s12943-023-01920-w (PMC10799368; doi:10.1186/s12943-023-01920-w)
Supplement: Supplementary file 11 — Additional 11: Supplementary Table S1. Full names and abbreviations of the tumor types involved in this study. [file 12943_2023_1920_MOESM11_ESM.docx]

| Abbreviation | Name | Normal | Tumor |
| --- | --- | --- | --- |
| ACC | Adrenocortical Carcinoma | 128 | 79 |
| BLCA | Bladder Urothelial Carcinoma | 28 | 408 |
| BRCA | Breast Invasive Carcinoma | 292 | 1098 |
| CESC | Cervical Squamous Cell Carcinoma and Endocervical Adenocarcinoma | 13 | 306 |
| CHOL | Cholangiocarcinoma | 9 | 36 |
| COAD | Colon Adenocarcinoma | 349 | 458 |
| ESCA | Esophageal Carcinoma | 664 | 162 |
| GBM | Glioblastoma Multiforme | 1157 | 167 |
| HNSC | Head and Neck Squamous Cell Carcinoma | 44 | 502 |
| KICH | Kidney Chromophobe | 52 | 65 |
| KIRC | Kidney Renal Clear Cell Carcinoma | 72 | 531 |
| KIRP | Kidney Renal Papillary Cell Carcinoma | 32 | 289 |
| LAML | Acute Myeloid Leukemia | 70 | 151 |
| LGG | Brain Lower Grade Glioma | 1157 | 525 |
| LIHC | Liver Hepatocellular Carcinoma | 160 | 373 |
| LUAD | Lung Adenocarcinoma | 347 | 515 |
| LUSC | Lung Squamous Cell Carcinoma | 49 | 501 |
| OV | Ovarian Serous Cystadenocarcinoma | 88 | 379 |
| PAAD | Pancreatic Adenocarcinoma | 171 | 178 |
| PRAD | Prostate Adenocarcinoma | 152 | 496 |
| READ | Rectum Adenocarcinoma | 10 | 167 |
| SKCM | Skin Cutaneous Melanoma | 813 | 471 |
| STAD | Stomach Adenocarcinoma | 206 | 375 |
| TGCT | Testicular Germ Cell Tumors | 165 | 156 |
| THCA | Thyroid Carcinoma | 337 | 510 |
| UCEC | Uterine Corpus Endometrial Carcinoma | 35 | 544 |
| UCS | Uterine Carcinosarcoma | 78 | 56 |

Supplementary Table 1: Full names and abbreviations of the tumor types involved in this study
