# Supplementary material for: Identification of HSP90B1 in pan-cancer hallmarks to aid development of a potential therapeutic target
Source: Mol Cancer. 2024 Jan 20;23:19. doi: 10.1186/s12943-023-01920-w (PMC10799368; doi:10.1186/s12943-023-01920-w)
Supplement: Supplementary file 12 — Additional 12: Supplementary Table S2. Analysis of potential anti-tumor drugs targeting HSP90B1-associated genes based on the CMap database.Each positive connectivity score signifies a positive correlation between the drug perturbation expression profile and the disease perturbation expression profile, which implies that the agent may induce or exacerbate the associated disease state. Conversely, each negative connectivity score indicates a negative correlation between the drug perturbation expression profile and the disease perturbation expression profile, suggesting that the agent may alleviate or even reverse the associated disease state. [file 12943_2023_1920_MOESM12_ESM.docx]

Supplementary Table 2: Analysis of potential anti-tumor drugs targeting HSP90B1-associated genes based on the CMap database

| Agent | Cell | Lable | Connectivity score |
| --- | --- | --- | --- |
| CCT-018159 | SKB | HSP inhibitor | -1.5886 |
| VER-155008 | A375 | HSP inhibitor | -1.4787 |
| VER-155008 | PC3 | HSP inhibitor | -1.3614 |
| tanespimycin | SW480 | HSP inhibitor | -1.361 |
| KW-2478 | HEK293 | HSP inhibitor | -1.35 |
| BIIB-021 | HT29 | HSP inhibitor | -1.3364 |
| tanespimycin | HCT116 | HSP inhibitor | -1.3046 |
| BIIB-021 | NPC | HSP inhibitor | -1.301 |
| KW-2478 | MCF7 | HSP inhibitor | -1.2574 |
| isogedunin | MCF7 | HSP inhibitor | -1.2253 |
| nomilin | HCC515 | HSP inhibitor | -1.2247 |
| BRD-A47144777 | A375 | HSP inhibitor | -1.2235 |
| nomilin | PC3 | HSP inhibitor | -1.2009 |
| XL-888 | A375 | HSP inhibitor | -1.1882 |
| SNX-2112 | A549 | HSP inhibitor | -1.1822 |
| isogedunin | HCC515 | HSP inhibitor | -1.1767 |
| VER-155008 | HEPG2 | HSP inhibitor | -1.0798 |
| VER-155008 | HCC515 | HSP inhibitor | -1.0746 |
| ganetespib | YAPC | HSP inhibitor | -1.0646 |
| VER-155008 | NCIH508 | HSP inhibitor | -1.059 |
| tanespimycin | A549 | HSP inhibitor | -1.0416 |
| BIIB-021 | MDAMB231 | HSP inhibitor | -1.0165 |
| VER-155008 | PHH | HSP inhibitor | -1.0043 |
| PU-H71 | NPC | HSP inhibitor | -0.9852 |
| VER-155008 | A549 | HSP inhibitor | -0.9841 |
| VER-155008 | HCT116 | HSP inhibitor | -0.9773 |
| VER-155008 | HA1E | HSP inhibitor | -0.9701 |
| alvespimycin | A375 | HSP inhibitor | -0.9425 |
| SNX-2112 | A375 | HSP inhibitor | -0.9149 |
| PU-H71 | PC3 | HSP inhibitor | -0.8698 |
| BIIB-021 | YAPC | HSP inhibitor | -0.8643 |
| isogedunin | A549 | HSP inhibitor | -0.8625 |
| isogedunin | A375 | HSP inhibitor | -0.8454 |
| alvespimycin | HEPG2 | HSP inhibitor | -0.8344 |
| AT-13387 | HA1E | HSP inhibitor | -0.8098 |
| alvespimycin | MCF7 | HSP inhibitor | -0.809 |
| alvespimycin | NPC | HSP inhibitor | -0.8014 |
| alvespimycin | JURKAT | HSP inhibitor | -0.7739 |
| XL-888 | U2OS | HSP inhibitor | -0.7728 |
| VER-155008 | NCIH596 | HSP inhibitor | -0.7538 |
| NVP-AUY922 | THP1 | HSP inhibitor | -0.7337 |
| ganetespib | PC3 | HSP inhibitor | -0.7335 |
| tanespimycin | HA1E | HSP inhibitor | -0.7295 |
| VER-155008 | MCF7 | HSP inhibitor | -0.727 |
| tanespimycin | THP1 | HSP inhibitor | -0.7255 |
| BIIB-021 | A375 | HSP inhibitor | -0.7244 |
| BIIB-021 | HEK293 | HSP inhibitor | -0.7182 |
| SNX-2112 | YAPC | HSP inhibitor | -0.706 |
| CCT-018159 | PHH | HSP inhibitor | -0.6919 |
| AT-13387 | PC3 | HSP inhibitor | -0.686 |
| CCT-018159 | A549 | HSP inhibitor | -0.6698 |
| BIIB-021 | ASC | HSP inhibitor | -0.6559 |
| alvespimycin | HELA | HSP inhibitor | -0.6551 |
| BRD-A47144777 | A549 | HSP inhibitor | -0.6339 |
| CCT-018159 | NPC | HSP inhibitor | -0.5895 |
| BRD-A47144777 | PC3 | HSP inhibitor | -0.5421 |
| tanespimycin | MCF10A | HSP inhibitor | -0.4722 |
| XL-888 | HUVEC | HSP inhibitor | 0.4118 |
| alvespimycin | HT29 | HSP inhibitor | 0.4282 |
| isogedunin | HT29 | HSP inhibitor | 0.5362 |
| BRD-A47144777 | MCF7 | HSP inhibitor | 0.5385 |
| VER-155008 | U937 | HSP inhibitor | 0.539 |
| tanespimycin | NPC | HSP inhibitor | 0.5689 |
| KW-2478 | HUVEC | HSP inhibitor | 0.5772 |
| BIIB-021 | SKB | HSP inhibitor | 0.5851 |
| tanespimycin | A375 | HSP inhibitor | 0.6095 |
| nomilin | HA1E | HSP inhibitor | 0.6197 |
| isogedunin | PC3 | HSP inhibitor | 0.667 |
| PU-H71 | SKB | HSP inhibitor | 0.6742 |
| VER-155008 | P1A82 | HSP inhibitor | 0.6773 |
| SNX-2112 | HUVEC | HSP inhibitor | 0.6781 |
| KW-2478 | A375 | HSP inhibitor | 0.6783 |
| tanespimycin | NEU | HSP inhibitor | 0.6857 |
| BRD-A47144777 | HT29 | HSP inhibitor | 0.6874 |
| XL-888 | HA1E | HSP inhibitor | 0.7113 |
| SNX-2112 | PC3 | HSP inhibitor | 0.7187 |
| CCT-018159 | A375 | HSP inhibitor | 0.7244 |
| tanespimycin | NOMO1 | HSP inhibitor | 0.7245 |
| BIIB-021 | HA1E | HSP inhibitor | 0.7296 |
| alvespimycin | YAPC | HSP inhibitor | 0.759 |
| XL-888 | HT29 | HSP inhibitor | 0.7959 |
| XL-888 | MCF7 | HSP inhibitor | 0.8147 |
| KW-2478 | MCF10A | HSP inhibitor | 0.834 |
| AT-13387 | A375 | HSP inhibitor | 0.8342 |
| CCT-018159 | HA1E | HSP inhibitor | 0.8357 |
| ganetespib | MCF7 | HSP inhibitor | 0.8643 |
| nomilin | VCAP | HSP inhibitor | 0.8782 |
| alvespimycin | HA1E | HSP inhibitor | 0.8788 |
| CCT-018159 | HT29 | HSP inhibitor | 0.8817 |
| VER-155008 | VCAP | HSP inhibitor | 0.8866 |
| SNX-2112 | MCF10A | HSP inhibitor | 0.8918 |
| NVP-AUY922 | NCIH508 | HSP inhibitor | 0.8959 |
| BIIB-021 | HELA | HSP inhibitor | 0.8972 |
| SNX-2112 | HEPG2 | HSP inhibitor | 0.9016 |
| CCT-018159 | ASC | HSP inhibitor | 0.9032 |
| NVP-AUY922 | MCF10A | HSP inhibitor | 0.9101 |
| KW-2478 | JURKAT | HSP inhibitor | 0.92 |
| AT-13387 | MCF10A | HSP inhibitor | 0.9481 |
| BIIB-021 | PC3 | HSP inhibitor | 0.9533 |
| XL-888 | HELA | HSP inhibitor | 0.954 |
| PU-H71 | HA1E | HSP inhibitor | 0.9659 |
| ganetespib | A375 | HSP inhibitor | 0.9664 |
| alvespimycin | PC3 | HSP inhibitor | 0.9675 |
| NVP-AUY922 | MDAMB231 | HSP inhibitor | 0.972 |
| BIIB-021 | MCF10A | HSP inhibitor | 0.9737 |
| CCT-018159 | PC3 | HSP inhibitor | 0.9748 |
| isogedunin | HA1E | HSP inhibitor | 0.975 |
| BIIB-021 | NEU | HSP inhibitor | 0.9797 |
| ganetespib | HT29 | HSP inhibitor | 0.9911 |
| NVP-AUY922 | A549 | HSP inhibitor | 0.9925 |
| tanespimycin | NCIH596 | HSP inhibitor | 0.9976 |
| VER-155008 | H1299 | HSP inhibitor | 1.0037 |
| CCT-018159 | U2OS | HSP inhibitor | 1.0105 |
| CCT-018159 | HCC515 | HSP inhibitor | 1.0166 |
| BIIB-021 | VCAP | HSP inhibitor | 1.0193 |
| PU-H71 | A375 | HSP inhibitor | 1.0247 |
| tanespimycin | U937 | HSP inhibitor | 1.0256 |
| PU-H71 | HCC515 | HSP inhibitor | 1.0413 |
| XL-888 | PC3 | HSP inhibitor | 1.0418 |
| tanespimycin | PC3 | HSP inhibitor | 1.0486 |
| VER-155008 | HT29 | HSP inhibitor | 1.0497 |
| isogedunin | VCAP | HSP inhibitor | 1.0536 |
| CCT-018159 | VCAP | HSP inhibitor | 1.054 |
| tanespimycin | HT29 | HSP inhibitor | 1.0552 |
| SNX-2112 | MCF7 | HSP inhibitor | 1.0571 |
| PU-H71 | A549 | HSP inhibitor | 1.0598 |
| BIIB-021 | HEPG2 | HSP inhibitor | 1.0696 |
| KW-2478 | PC3 | HSP inhibitor | 1.08 |
| SNX-2112 | HT29 | HSP inhibitor | 1.0936 |
| tanespimycin | AGS | HSP inhibitor | 1.0955 |
| SNX-2112 | HELA | HSP inhibitor | 1.098 |
| PU-H71 | NEU | HSP inhibitor | 1.0994 |
| radicicol | PHH | HSP inhibitor | 1.1039 |
| CCT-018159 | MCF7 | HSP inhibitor | 1.1155 |
| XL-888 | MDAMB231 | HSP inhibitor | 1.12 |
| tanespimycin | NCIH2073 | HSP inhibitor | 1.1283 |
| KW-2478 | HA1E | HSP inhibitor | 1.1312 |
| PU-H71 | ASC | HSP inhibitor | 1.1454 |
| KW-2478 | HELA | HSP inhibitor | 1.1671 |
| KW-2478 | A549 | HSP inhibitor | 1.1772 |
| KW-2478 | YAPC | HSP inhibitor | 1.1835 |
| NVP-AUY922 | PC3 | HSP inhibitor | 1.1872 |
| tanespimycin | H1299 | HSP inhibitor | 1.2035 |
| tanespimycin | VCAP | HSP inhibitor | 1.2038 |
| PU-H71 | PHH | HSP inhibitor | 1.2183 |
| alvespimycin | A375 | HSP inhibitor | 1.2202 |
| ganetespib | HA1E | HSP inhibitor | 1.2297 |
| VER-155008 | NCIH2073 | HSP inhibitor | 1.2352 |
| KW-2478 | U2OS | HSP inhibitor | 1.2447 |
| BIIB-021 | MCF7 | HSP inhibitor | 1.2449 |
| ganetespib | HELA | HSP inhibitor | 1.2472 |
| radicicol | A549 | HSP inhibitor | 1.2654 |
| tanespimycin | HEPG2 | HSP inhibitor | 1.273 |
| alvespimycin | NEU | HSP inhibitor | 1.2819 |
| NVP-AUY922 | SKBR3 | HSP inhibitor | 1.2841 |
| BIIB-021 | HCC515 | HSP inhibitor | 1.2901 |
| alvespimycin | ASC | HSP inhibitor | 1.2931 |
| KW-2478 | THP1 | HSP inhibitor | 1.2932 |
| PU-H71 | HT29 | HSP inhibitor | 1.2938 |
| alvespimycin | A549 | HSP inhibitor | 1.3 |
| BIIB-021 | THP1 | HSP inhibitor | 1.3033 |
| VER-155008 | THP1 | HSP inhibitor | 1.3103 |
| CCT-018159 | HEPG2 | HSP inhibitor | 1.325 |
| radicicol | SKB | HSP inhibitor | 1.3324 |
| SNX-2112 | HEK293 | HSP inhibitor | 1.3358 |
| alvespimycin | A549 | HSP inhibitor | 1.3449 |
| radicicol | U2OS | HSP inhibitor | 1.3609 |
| NVP-AUY922 | VCAP | HSP inhibitor | 1.3688 |
| NVP-AUY922 | NOMO1 | HSP inhibitor | 1.3688 |
| alvespimycin | SKB | HSP inhibitor | 1.3779 |
| SNX-2112 | HA1E | HSP inhibitor | 1.3826 |
| XL-888 | A549 | HSP inhibitor | 1.3866 |
| tanespimycin | HCC515 | HSP inhibitor | 1.3916 |
| BRD-A47144777 | VCAP | HSP inhibitor | 1.3968 |
| radicicol | MCF7 | HSP inhibitor | 1.3976 |
| NVP-AUY922 | U937 | HSP inhibitor | 1.3996 |
| alvespimycin | HA1E | HSP inhibitor | 1.4048 |
| radicicol | HA1E | HSP inhibitor | 1.4221 |
| PU-H71 | HEPG2 | HSP inhibitor | 1.4369 |
| AT-13387 | YAPC | HSP inhibitor | 1.4387 |
| AT-13387 | HEK293 | HSP inhibitor | 1.443 |
| PU-H71 | VCAP | HSP inhibitor | 1.4432 |
| SNX-2112 | MDAMB231 | HSP inhibitor | 1.4668 |
| radicicol | HEPG2 | HSP inhibitor | 1.4759 |
| AT-13387 | MCF7 | HSP inhibitor | 1.4848 |
| PU-H71 | MCF7 | HSP inhibitor | 1.4979 |
| BRD-A47144777 | HCC515 | HSP inhibitor | 1.5048 |
| AT-13387 | HELA | HSP inhibitor | 1.5222 |
| NVP-AUY922 | HS578T | HSP inhibitor | 1.5326 |
| VER-155008 | AGS | HSP inhibitor | 1.549 |
| BIIB-021 | A549 | HSP inhibitor | 1.5553 |
| NVP-AUY922 | SW480 | HSP inhibitor | 1.5698 |
| NVP-AUY922 | AGS | HSP inhibitor | 1.5714 |
| XL-888 | YAPC | HSP inhibitor | 1.5744 |
| radicicol | ASC | HSP inhibitor | 1.5815 |
| tanespimycin | PHH | HSP inhibitor | 1.5834 |
| radicicol | NEU | HSP inhibitor | 1.5885 |
| tanespimycin | MCF7 | HSP inhibitor | 1.5886 |
| tanespimycin | NCIH508 | HSP inhibitor | 1.5902 |
| tanespimycin | P1A82 | HSP inhibitor | 1.5907 |
| NVP-AUY922 | HCT116 | HSP inhibitor | 1.6028 |
| alvespimycin | HT29 | HSP inhibitor | 1.6051 |
| AT-13387 | HT29 | HSP inhibitor | 1.621 |
| radicicol | A375 | HSP inhibitor | 1.6271 |
| alvespimycin | PHH | HSP inhibitor | 1.6331 |
| radicicol | VCAP | HSP inhibitor | 1.6384 |
| alvespimycin | VCAP | HSP inhibitor | 1.6461 |
| radicicol | HCC515 | HSP inhibitor | 1.6484 |
| NVP-AUY922 | NCIH596 | HSP inhibitor | 1.6534 |
| alvespimycin | MCF7 | HSP inhibitor | 1.6541 |
| KW-2478 | MDAMB231 | HSP inhibitor | 1.6599 |
| ganetespib | U2OS | HSP inhibitor | 1.6756 |
| SNX-2112 | HCC515 | HSP inhibitor | 1.6905 |
| KW-2478 | HT29 | HSP inhibitor | 1.7117 |
| XL-888 | HEK293 | HSP inhibitor | 1.7361 |
| BIIB-021 | JURKAT | HSP inhibitor | 1.7481 |
| NVP-AUY922 | NCIH2073 | HSP inhibitor | 1.7559 |
| alvespimycin | HCC515 | HSP inhibitor | 1.7851 |
| NVP-AUY922 | HEPG2 | HSP inhibitor | 1.787 |
| alvespimycin | PC3 | HSP inhibitor | 1.7934 |
| XL-888 | MCF10A | HSP inhibitor | 1.8188 |
| tanespimycin | U2OS | HSP inhibitor | 1.8193 |
| radicicol | PC3 | HSP inhibitor | 1.8197 |
| NVP-AUY922 | HA1E | HSP inhibitor | 1.8763 |
| radicicol | HT29 | HSP inhibitor | 1.9104 |
| NVP-AUY922 | MCF7 | HSP inhibitor | 1.9262 |
| NVP-AUY922 | H1299 | HSP inhibitor | 1.9283 |
| NVP-AUY922 | A375 | HSP inhibitor | 1.9361 |
| ganetespib | A549 | HSP inhibitor | 2.0018 |
| BRD-A47144777 | U2OS | HSP inhibitor | 2.018 |
| NVP-AUY922 | HT29 | HSP inhibitor | 2.0397 |
| NVP-AUY922 | HCC515 | HSP inhibitor | 2.0474 |
| NVP-AUY922 | U2OS | HSP inhibitor | 2.0897 |

Footnote: Each positive connectivity score signifies a positive correlation between the drug perturbation expression profile and the disease perturbation expression profile, which implies that the agent may induce or exacerbate the associated disease state. Conversely, each negative connectivity score indicates a negative correlation between the drug perturbation expression profile and the disease perturbation expression profile, suggesting that the agent may alleviate or even reverse the associated disease state.
